# Macrofungal Diversity and Distribution Patterns in the Primary Forests of the Shaluli Mountains

**DOI:** 10.3390/jof9040491

**Published:** 2023-04-19

**Authors:** Xixi Han, Dongmei Liu, Mingzhe Zhang, Maoqiang He, Jiaxin Li, Xinyu Zhu, Meiqi Wang, Naritsada Thongklang, Ruilin Zhao, Bin Cao

**Affiliations:** 1 State Key Laboratory of Mycology, Institute of Microbiology, Chinese Academy of Sciences, Beijing 100101, China; 2 Center of Excellence in Fungal Research, Mae Fah Luang University, Chiang Rai 57100, Thailand; 3 Institue of Ecology, Chinese Research Academy of Environmental Sciences, Beijing 100012, China; 4 College of Life Sciences, University of Chinese Academy of Sciences, Beijing 100049, China

**Keywords:** Shaluli Mountains, macrofungal diversity, community composition, vegetation type, vertical distribution

## Abstract

The Shaluli Mountains are located in the southeastern part of the Tibetan Plateau at an elevation of 2500–5000 m. They are characterized by a typical vertical distribution of climate and vegetation and are considered a global biodiversity hotspot. We selected ten vegetation types at different elevation gradients representing distinct forests in the Shaluli Mountains to assess the macrofungal diversity, including subalpine shrub, *Pinus* spp., *Populus* spp., *Pinus* spp. and *Quercus* spp., *Quercus* spp., *Abies* spp., *Picea* spp. and *Abies* spp., *Picea* spp., *Juniperus* spp., and alpine meadow. In total, 1654 macrofungal specimens were collected. All specimens were distinguished by morphology and DNA barcoding, resulting in the identification of 766 species belonging to 177 genera in two phyla, eight classes, 22 orders, and 72 families. Macrofungal species composition varied widely among vegetation types, but ectomycorrhizal fungi were predominant. In this study, the analysis of observed species richness, the Chao1 diversity index, the invsimpson diversity index, and the Shannon diversity index revealed that the vegetation types with higher macrofungal alpha diversity in the Shaluli Mountains were composed of *Abies*, *Picea*, and *Quercus*. The vegetation types with lower macrofungal alpha diversity were subalpine shrub, *Pinus* spp., *Juniperus* spp., and alpine meadow. The results of curve-fitting regression analysis showed that macrofungal diversity in the Shaluli Mountains was closely related to elevation, with a trend of increasing and then decreasing with rising elevation. This distribution of diversity is consistent with the hump-shaped pattern. Constrained principal coordinate analysis based on Bray–Curtis distances indicated that macrofungal community composition was similar among vegetation types at similar elevations, while vegetation types with large differences in elevation differed significantly in macrofungal community composition. This suggests that large changes in elevation increase macrofungal community turnover. This study is the first investigation of the distribution pattern of macrofungal diversity under different vegetation types in high-altitude areas, providing a scientific basis for the conservation of macrofungal resources.

## 1. Introduction

Fungi are among the most species-rich taxa in the terrestrial biosphere [1], and their key members, the macrofungi, are of high economic value [2], and play an important role in material cycling, energy flow, and plant community succession of forest ecosystems [3,4]. Among them, mycorrhizal fungi can form symbioses with the majority of terrestrial plant roots. These symbioses promote plant growth and development, protect ecologically sensitive areas from soil erosion, and maintain ecosystem stability [5,6,7]. Many species of macrofungi are important for food and medicine, such as *Agaricus bisporus* (J.E. Lange) Imbach, *Tricholoma atrosquamosum* Sacc., and *Hericium erinaceus* (Bull.) Pers [8]. They are not only high in protein and low in fat, but also produce polysaccharides, polysaccharide proteins, polysaccharide peptides, triterpenoids, sterols, nucleosides, and other active ingredients that have anti-cancer, anti-tumor, blood pressure-lowering, blood lipid-lowering, blood sugar-lowering, and immunomodulatory effects [2,9,10]. There are also some macrofungi that have toxic properties, such as *Amanita parvipantherina* (Zhu L. Yang, M. Weiss & Oberw.), *Gyromitra infula* (Schaeff.) Quél., and *Panaeolus fimicola* (Pers.) Gillet [11]. Due to their high adaptability and survival rates, macrofungi are widely distributed in environments such as forests, shrublands, grasslands, and urban areas [12,13,14]. However, macrofungal diversity and community composition vary considerably between habitats [15,16,17]. Moreover, diversity is also moderated by climate, soil parameters, anthropogenic disturbances (trampling, slash, and burn), and other factors. This variation mainly depends on vegetation type, substrate and habitat elevation, as well as latitude and longitude [18,19,20,21,22]. While studies on macrofungal species diversity in specific regions were extensively conducted [23,24,25], research on the vertical distribution patterns of macrofungi and their formation mechanisms is still ongoing [26,27,28,29].

The rapid uplift of the Tibetan Plateau during the plate collision period, the rapid formation of the Transverse Range, as well as the advance and retreat of Quaternary glaciers, not only resulted in intense plant and animal population evolution but also had far-reaching effects on fungal populations and evolution in this region [30,31,32]. The Tibetan Plateau ecosystems have a distinct distribution pattern, influenced by topography, atmospheric circulation, and land–sea distribution, with a range of climate types from low to high altitudes, including tropical, subtropical, and temperate. [33,34,35]. The vegetation ecosystem types can be classified from bottom to top as arid valley scrub, low mountain moist forest, subalpine wet forest, subalpine moist forest, subalpine arid scrub, and alpine moist tundra [36,37,38]. Due to geological and historical activities, climatic variations, topographical environmental conditions, and the great variety of flora types, this region is home to many macrofungal species that are distinctive and rich in diversity. The pattern of biodiversity along environmental gradients is a critical scientific issue in biodiversity research [39], and species diversity is the simplest and most effective way to describe the diversity of communities and regions, which is the essence of biodiversity [40]. It was demonstrated that the diversity of biome species is characterized by five major patterns of variation with elevation gradient: hump-shaped [41], negatively correlated [42], concave-shaped [43], positively correlated [44], and uncorrelated [45]. Liu (2015) proposed that the general pattern of species diversity distribution along the elevation gradient on the Tibetan Plateau is a hump-shaped distribution [41]. This argument was supported by research on plants [44,46], birds [47], fish [48], insects [26], mammals [49] and bacteria [50], while the distribution pattern of macrofungal species diversity in this area was not investigated.

The Shaluli Mountains are part of the Hengduan Mountains and are located in the southeast of the Tibetan Plateau. They have an average elevation of over 3000 m and range in height from 2500 to 5000 m [51,52]. In this study, 10 vegetation types at different elevations in the primary forest of the Shaluli Mountains were selected to investigate the species’ diversity and community composition of macrofungi. We aimed to answer the following scientific questions: What is the species composition of macrofungi in the primary forest of the Shaluli Mountains? How does macrofungal species’ diversity and community composition vary between vegetation types? What is the pattern of distribution of macrofungal species diversity along the elevational gradient, is it a hump-shaped pattern?

## 2. Materials and Methods

### 2.1. Sample Plot Setup and Sporocarp Sampling

**Figure 1:**
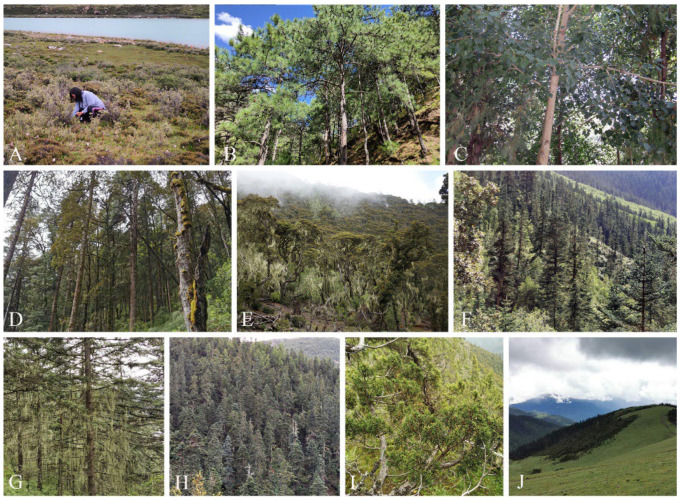
Vegetation types in the Shaluli Mountains. (**A**) SS; (**B**) Pin; (**C**) Pop; (**D**) PQ; (**E**) Que; (**F**) Abi; (**G**) PA; (**H**) Pic; (**I**) Jun; (**J**) AM.

**Figure 2:**
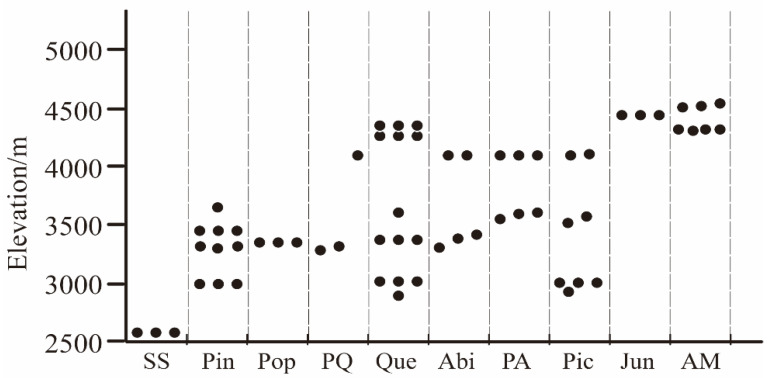
Sample plots distribution and elevation. The horizontal axis is the vegetation type, arranged from left to right according to the average elevation from low to high.

There are five vegetation belts in the Shaluli Mountains, from bottom to top: arid chaparral scrub belt, semi-arid scrub and semi-humid coniferous forest belt, spruce forest belt, fir forest belt, and alpine scrub-meadow belt [36]. We selected the main vegetation types in each vegetation belt and established at least three sample plots in each vegetation type. Ten vegetation types with different elevation gradients were selected in the Shaluli Mountains to assess macrofungal diversity, including subalpine shrub (SS), *Pinus* spp. (Pin), *Populus* spp. (Pop), *Pinus* spp. and *Quercus* spp. (PQ), *Quercus* spp. (Que), *Abies* spp. (Abi), *Picea* spp. and *Abies* spp. (PA), *Picea* spp. (Pic), *Juniperus* spp. (Jun), and alpine meadow (AM), with 20 m × 20 m sample plots under each vegetation type (Figure 1). We ensured that three sample plots were set up under each vegetation type, and when a vegetation type was distributed at different elevations, we would set up additional sample plots at different elevations, the exact number of additional plots will be determined according to the field situation, for a total of 62 plots (Figure 2).

We collected all macrofungi, including those growing on tree and dung as well as on the ground, within the sample plots twice during the rainy seasons of 2019 and 2020, in August. Photographs were taken of the habitat vegetation, growing substrate, and morphological characteristics of the specimens. The number of specimens from each sample plot were counted. A dryer was used to completely dry fresh specimens, which are now conserved in the Herbarium Mycologicum Academiae Sinicae, Beijing, China (HMAS).

### 2.2. Species Identification

We conducted morphological observations on all macrofungal specimens. Macroscopic features of the specimens included the pileus, lamellae, stipe, and ring of macrofungus. The protocols for the morphological analysis all followed Largent’s method [53].

DNA was extracted from dried specimens using a Broad-spectrum Plant Rapid Genomic DNA Kit (Biomed), following the manufacturer’s instructions. Final elutions were performed in a total volume of 100 µL. Primers ITS4 and ITS5 were used for the nuclear internal transcribed spacer (nrITS) of the rDNA region [54]. PCR was performed in 25 µL reactions consisting of 2 µL genomic DNA, 1 µL upstream and 1 µL downstream primers, 9 µL ddH2O, and 12 µL 2 × Es Taq MasterMix (Beijing Cowin Biotech Co., Ltd., Beijing, China). Then, we performed under the following conditions: 94 °C for 5 min, followed by 30 cycles of 94 °C for 30 s, 55 °C for 40 s, 72 °C for 50 s, and a final extension step at 72 °C for 10 min before storage at 12 °C [55,56]. The PCR products were detected by electrophoresis and sent to BGI Genomics Co., Ltd., Shenzhen, China, for purification and sequencing [57].

ITS is a standard barcode marker for fungi, with 97% to 99% sequence identity in this region, and is commonly used to delimit species [58,59]. We compared the obtained ITS sequences with those in the NCBI database and considered the same species with more than 99% identity, 97–99% identity as a close species (cf.), and less than 97% identity as a species of that genus (sp.). Specimens for which ITS sequences were not successfully obtained were distinguished from species based on their morphology only. Species identification was performed by ITS barcoding in combination with morphological analysis, and species differentiation was performed for macrofungi that were temporarily difficult to identify at the species level, such as *Russula* sp1, *Russula* sp2, etc. The classification of Basidiomycota was based on He [60], and the classification of Ascomycota with reference to Wijayawardene [61].

### 2.3. Statistical Analysis

Statistical analysis and visualization of the data were performed using R version 4.4.1, Python version 3.9.7, and TBtools version 1.098761 [62,63]. All macrofungal specimens identified in the sample plots, as well as the number of individuals collected, were initially recorded using Excel (2019). UpSet plots were used to show species overlap and endemism among the ten vegetation types [64,65]. The diversity index calculation was performed by the “diversity” and “plyr” functions in the “vegan” package [66]. Macrofungal alpha diversity was characterized using the observed species richness, Chao1 diversity index, Shannon diversity index, and invsimpson diversity index calculated by the diversity command [67,68]. To investigate the variation of macrofungal species diversity along the elevation gradient, the curve-fitting regression of observed species richness with elevation was analyzed using the ggtrendline package.

Due to topographic and elevation differences, the number of sample plots established for each of the 10 vegetation types varied. To avoid the effect of different numbers of sample plots for different vegetation types impacting the diversity results, this study used repeated draws to calculate alpha diversity and took the mean value to correct for alpha diversity [69]. We randomly selected three samples from each vegetation type at a time to calculate alpha diversity, and the number of replicate draws was increased at a frequency of 100 until a stable mean value was obtained. The Kruskal–Wallis test (KW) was employed to identify differences in macrofungal alpha diversity among vegetation types. If significant differences (*p* < 0.05) were observed by KW, multiple comparisons between means were performed using Dunn’s test.

Beta-diversity analysis was used to compare differences in community composition between samples. To assess the effect of different vegetation types on macrofungal community composition, beta diversity (diversity between samples) was compared using Bray–Curtis distances and constrained principal coordinate analysis (CPCoA) [70,71]. Unless stated otherwise, statistical analyses were performed in R version 4.4.1. 

## 3. Results

### 3.1. Macrofungal Composition

A total of 1654 specimens were collected from 62 sample plots. They were identified as 766 species, belonging to two phyla, eight classes, 22 orders, 72 families, and 177 genera (Table 1). Dividing by phyla, 728 species of Basidiomycota and 38 species of Ascomycota were identified, representing 95% and 5% of the specimens belonging to each phylum, respectively. Divided by class, the species of Agaricomycetes (718 species, 93.7%), Pezizomycetes (27 species, 3.5%), and Dacrymycetes (8 species, 1%) accounted for a relatively large number of specimens collected. As for the order of macrofungi, Agaricales (493 species, 64.3%), Russulales (107 species, 13.9%), and Boletales (46 species, 6%) accounted for the majority of the species (Table S1).

Among the identified species, there were 19 dominant families (number of species ≥ 10 species) of macrofungi (Table 2). The Cortinariaceae was the most diverse family. In addition, 53 families contained less than 10 species, accounting for 73.61% of the families and 22.85% of the identified species (Table S1).

**Figure 3:**
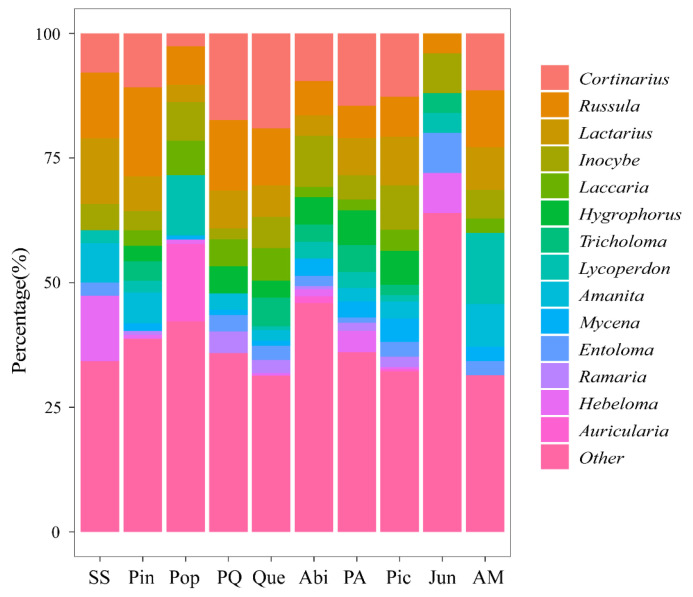
Bar chart of the major genera of macrofungal communities by vegetation type. The horizontal axis is the vegetation type, arranged from left to right according to the average elevation from low to high.

**Figure 4:**
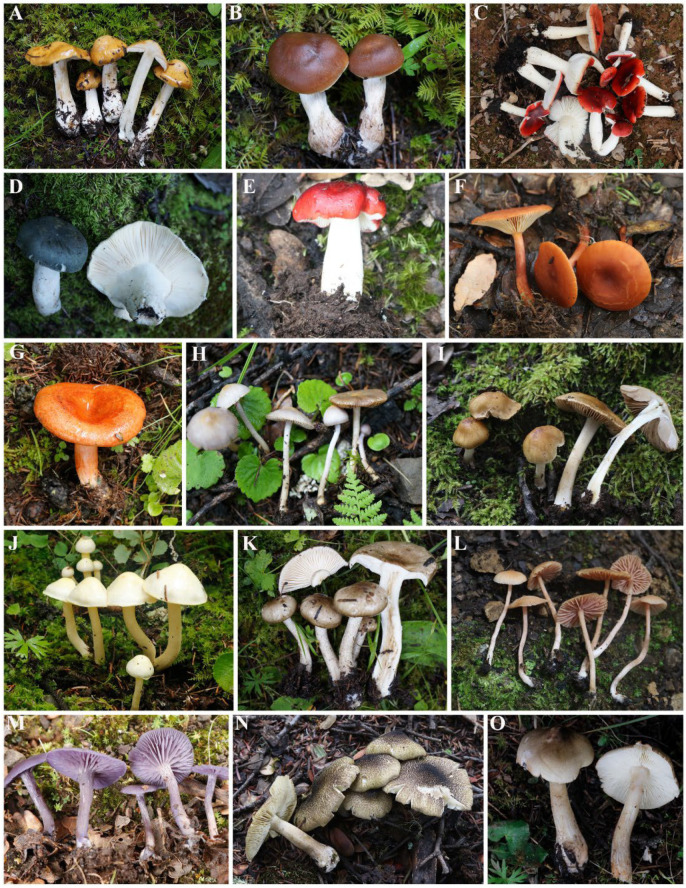
Photos of dominant genera. (**A**) *Cortinarius aurantionapus*; (**B**) *C. illuminus*; (**C**) *Russula* cf. *emetica*; (**D**) *R.* cf. *faustiana*; (**E**) *R.* cf. *emetica*; (**F**) *Lactarius alpinihirtipes*; (**G**) *L. aurantiosordidus*; (**H**) *Inocybe geophylla*; (**I**) *I.* cf. *ceskae*; (**J**) *Hygrophorus* cf. chrysodon; (**K**) *Hygrophorus* cf. *agathosmus*; (**L**) *Laccaria bicolor*; (**M**) *L. moshuijun*; (**N**) *Tricholoma atrosquamosum*; (**O**) *T. saponaceum*.

Among the identified species, there were 16 dominant genera (number of species ≥ 10 species) of macrofungi (Table 3). The *Cortinarius*, *Russula*, and *Inocybe* were the most diverse genera. In addition, 66 genera contained 2–9 species, accounting for 37.29% of the genera and 27.55% of the identified species; 95 of the genera contained only one species, accounting for 53.67% of the genera and 12.40% of the identified species (Table S1). The main constituent genera of each vegetation type are shown in Figure 3, and select morphological maps of the main genera are shown in Figure 4, as detailed in Table S1.

**Figure 5:**
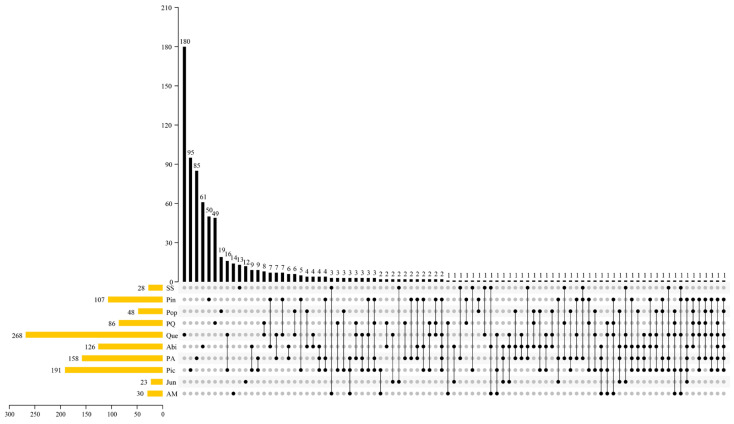
UpSet plots were used to display the overlap between ten vegetation types of macrofungal species. The horizontal bars indicate the number of macrofungal species in each vegetation type, with 28, 107, 48, 86, 268, 126, 158, 191, 23, and 30 species identified in SS, Pin, Pop, PQ, Que, Abi, PA, Pic, Jun, and AM, respectively. The vertical bars display the number of unique and shared species. Specifically, 13, 50, 19, 49, 180, 61, 85, 95, 12, and 14 species were identified as endemic to each vegetation type and found only in that type.

According to Figure 5, the majority of macrofungi were found in a single vegetation type, and the most widely distributed species in the area were *Armillaria cepistipes* Velen. and *Cortinarius* sp1. *Armillaria cepistipes* was observed in six vegetation types, namely Pin, Pop, Que, Abi, PA, and Pic, while *Cortinarius* sp1 was present in six vegetation types: Pin, PQ, Que, Abi, PA, and Pic.

### 3.2. Macrofungal Alpha Diversity

**Figure 6:**
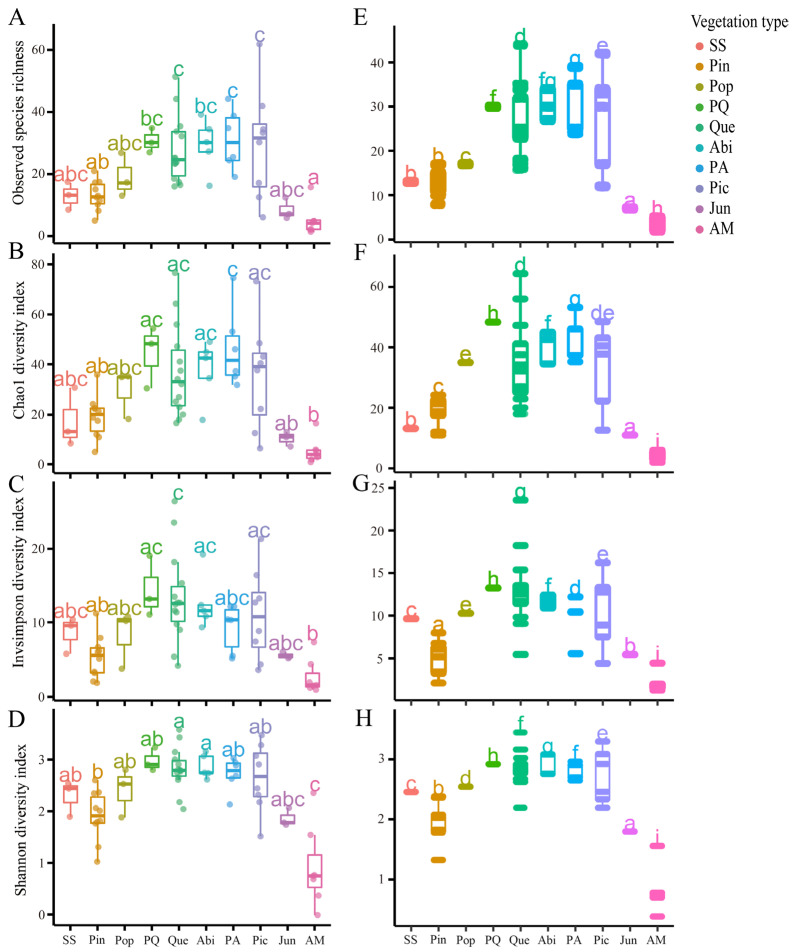
Alpha diversity analysis based on different vegetation types. Observed species richness (**A**), Chao1 diversity index (**B**), invsimpson diversity index (**C**), Shannon diversity index(**D**); post-correction observed species richness (**E**); post-correction Chao1 diversity index (**F**), post-correction invsimpson diversity index (**G**); post-correction Shannon diversity index (**H**). The box extends from the 25th to the 75th percentile, with the central line in each box representing the median value of the data set. Significance was determined by the Kruskal–Wallis test with the Dunn’s multiple comparison test. (*p* < 0.05). The sample sizes are as follows: (**A**–**D**): SS (*n* = 3), Pin (*n* = 10), Pop (*n* = 3), PQ (*n* = 3), Que (*n* = 14), Abi (*n* = 5), PA (*n* = 6), Pic (*n* = 8), Jun (*n* = 3), AM (*n* = 7); (**E**–**H**): SS (*n* = 1000), Pin (*n* = 1000), Pop (*n* = 1000), PQ (*n* = 1000), Que (*n* = 1000), Abi (*n* = 1000), PA (*n* = 1000), Pic (*n* = 1000), Jun (*n* = 1000), AM (*n* = 1000). The horizontal axis is the vegetation type, arranged from left to right according to the average elevation from low to high.

We used four diversity indices to assess the alpha diversity of macrofungi in the different vegetation types. The observed species richness and Chao1 diversity index showed that macrofungal species richness was significantly lower in AM than in PQ, Que, Abi, PA, and Pic (Figure 6A,B). The observed species richness showed that Pin was significantly lower than Que, PA, and Pic (Figure 6A). Chao1 diversity index suggests that Pin is significantly less rich than PA (Figure 6B).

The invsimpson diversity index showed that AM was significantly lower than that of Pin, Que, AP, and Picea spp (Figure 6C). The Shannon diversity index showed that AM was significantly lower than the other eight vegetation types except for Jun (Figure 6D). Both the invsimpson diversity index and the Shannon diversity index showed that the macrofungal diversity of Pin was significantly lower than that of Que (Figure 6C,D).

**Figure 7:**
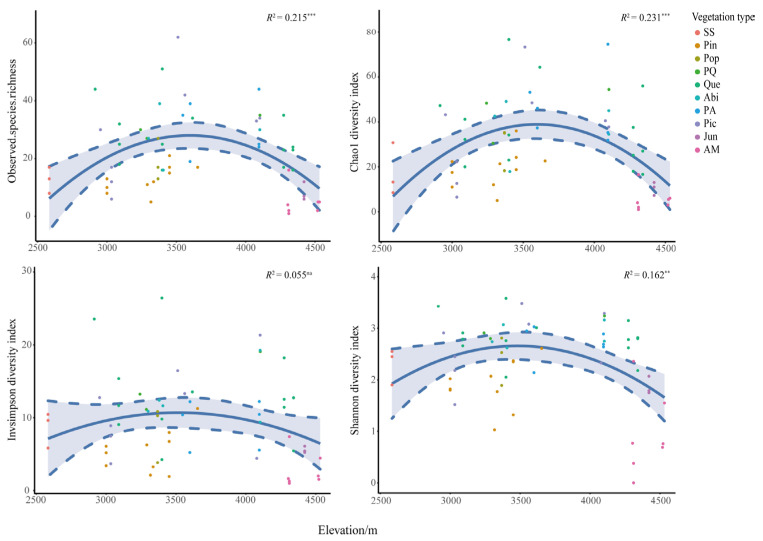
Variation of macrofungal observed species richness along the elevation gradient. *R*^2^, adjustment r squared of the regression equation; na, *p* value of the regression equation (*p*) is greater than 0.05; **, *p* is less than 0.01; ***, *p* is less than 0.001; gray areas are the 95% confidence intervals. Each point corresponds to a different sample with colors indicating vegetation type.

The alpha diversity of macrofungi was corrected by repeated sampling of plots, and the results showed significant differences in macrofungal species richness and diversity between vegetation types (Figure 6E–H). The ten vegetation types included in this study, SS, Pin, Pop, PQ, Que, Abi, PA, Pic, Jun, and AM, were located at gradually increasing elevations (Figure 2), where their macrofungal alpha diversity tended to increase and then decrease with increasing elevation (Figure 7). It was also found that the alpha diversity of macrofungi was higher in vegetation types containing *Picea*, *Abies*, and *Quercus* genera than in other vegetation types. In contrast, the macrofungal diversity of four vegetation types, SS, Pin, Jun, and AM, was relatively low.

### 3.3. Macrofungal Beta Diversity

**Figure 8:**
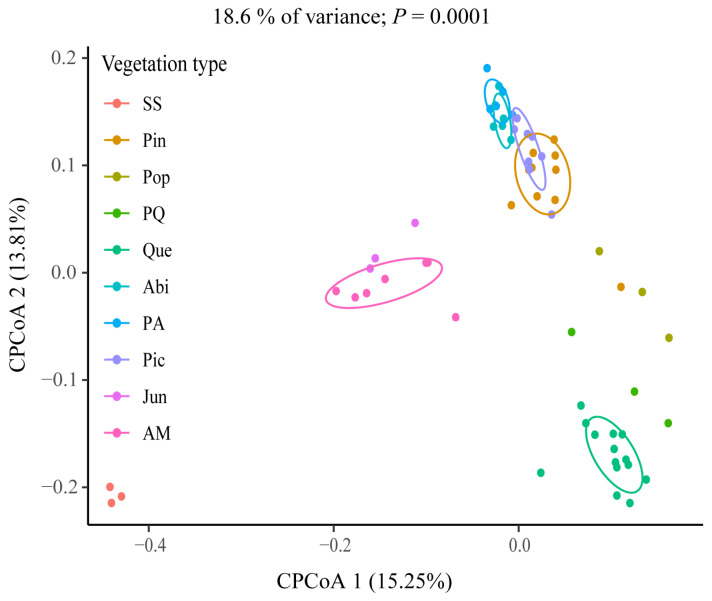
Constrained PCoA plot of Bray–Curtis distances constrained by vegetation type (18.6% of variance explained, *p* = 0.001; *n* = 62). Each point corresponds to a different sample with colors indicating vegetation type. The percentage of variation indicated on each axis corresponds to the proportion of the total variance explained by the projection.

To assess the effect of different vegetation on macrofungal community composition, we compared β-diversity and macrofungal community composition between vegetation types using Bray–Curtis distances and constrained principal coordinate analysis (CPCoA) (Figure 8). The results showed some similarity in the macrofungal community composition of Jun and AM, as well as Pin, Abi, PA, and Pic, indicating similar macrofungal community composition of vegetation corresponded with similar elevation distribution. The macrofungal community composition of the four groups of vegetation types: SS; Pin, Abi, PA, and Pic; Que; Jun and AM were significantly different, indicating that the macrofungal community composition of vegetation with large elevation variation was significantly different.

## 4. Discussion

### 4.1. Macrofungal Species Diversity Composition

This is the first systematic study on the diversity of macrofungi under typical vegetation types of the Shaluli Mountains in the southern part of the Transverse Ranges in China. The results indicated that macrofungi are abundant in the Shaluli Mountains at an average elevation of 3000 m above sea level. The species composition is dominated by fungi of the phylum Basidiomycota, especially the orders Agaricales, Russulales, Boletales, and Gomphales. In this study, 178 genera of macrofungi were identified, among which the more species-rich genera were *Cortinarius*, *Russula*, *Inocybe*, *Laccaria*, and *Tricholoma* (Figure 3). The macrofungi of the Shaluli Mountains were dominated by ectomycorrhizal fungi (64.1%), followed by saprophytic fungi (27.6%), in addition to 8.3% of species with an unknown trophic mode. The geographic composition of the more species-rich genera is dominated by components widespread throughout the world, such as *Cortinarius*, *Russula*, and *Inocybe*. This is followed by the northern temperate components, such as *Lactarius* [60,61].

### 4.2. Macrofungal Species Conservation

In 2016, the “Red List Assessment of Macrofungi in China” project was launched to evaluate the threatened status of macrofungi nationwide [72,73]. Macrofungal experts from across China were mobilized and organized to assess the threatened status of 9302 macrofungal species reported in China [74]. Of the 766 macrofungal species identified in this study, only 227 species were assessed [72]. Among them, there were three species of vulnerable (VU) macrofungi, including *Naematelia aurantialba* (Bandoni & M. Zang) Millanes & Wedin, *Hericium erinaceus* (Bull.) Pers., and *Tricholoma matsutake* (S. Ito & S. Imai) Singer. These three threatened macrofungi are distributed in Pin and Que, and they are all edible and medicinal fungi with high economic value [11,75]. Therefore, we propose to strengthen the protection of these two vegetation types in order to avoid the reduction in and destruction of the habitats of threatened macrofungal species.

### 4.3. Correlation between Macrofungal Diversity and Vegetation Types

At the local scale, fungal diversity and community composition are strongly correlated with elevation, but the main driver of diversity is vegetation type. Previous research indicated examples of the importance of vegetation type, such that the macrofungi present can even be specific to the species of tree [19,22,28]. This study showed that there were large differences in the composition of macrofungal species under different vegetation types in the Shaluli Mountains (Figure 3). In fact, 75% of the macrofungal species were collected under only one single vegetation type (Figure 4), with a certain proportion of endemic macrofungal species present in each vegetation type [76,77].

For macrofungal diversity under different vegetation types, O’Hanlon investigated macrofungal diversity in four vegetation types in Ireland, including ash, oak, Scots pine and Sitka spruce, and found significantly higher macrofungal species richness in Sitka spruce (coniferous forests) than in ash (deciduous forests) [77]. In contrast, in the temperate Wunvfeng National Forest Park in China, macrofungal diversity increased with the amount of *Quercus mongolica* (deciduous tree) in the forest [78]. However, in the Western Carpathians of Slovakia, macrofungal diversity analysis showed a higher species richness under beech (deciduous forests) than under spruce (coniferous forests) [27]. Despite living in the same climatic zone, macrofungal alpha diversity varied considerably under different vegetation types. This study showed that the vegetation types with high alpha diversity of macrofungi in the Shaluli Mountains were composed of *Abies*, *Picea*, and *Quercus*. Lower alpha diversity was found in the SS, Pin, Jun, and AM. In addition, although Abi, Pic, and Pin were coniferous forests, the alpha diversity of Pin was significantly lower than that of the other two stands (Figure 6). Que and Pop are both deciduous forests, but the alpha diversity of the former is significantly higher than that of the latter (Figure 6E–H). The above results suggest that macrofungal alpha diversity analysis should be conducted by vegetation type in a region with a large elevation span and rich vegetation types, in order to capture macrofungal diversity accurately and completely in a comprehensive manner.

### 4.4. Correlation between Macrofungal Diversity Patterns and Elevation

Previous research suggested that macrofungal diversity and elevation are closely related [17]. Fungal diversity decreased with elevation in the 0–400 m elevation range of Byeonsanbando National Park, Korea [79]. Similarly, macrofungal and ectomycorrhizal fungal diversity decreased with elevation in the range of 2700–3400 m in the Lijiang Subalpine Botanical Garden [80]. However, coniferous forests, deciduous forests, and scrubs were found to have an increasing and then decreasing distribution pattern of the arbuscular mycorrhizal fungi under the vegetation types of an elevation gradient from 100 m to 2300 m in central Japan [81]. The results of alpha diversity analysis in this study showed that macrofungal diversity in the Shaluli Mountains system was closely related to altitude. It showed a trend of increasing diversity at moderate heights and then decreasing diversity as the elevation increased, such that its vertical distribution pattern was consistent with the hump-shaped pattern [82]. Constrained principal coordinate analysis based on Bray–Curtis distances indicated that the macrofungal species composition was similar among vegetation types at similar elevations, while significant changes in macrofungal community composition was observed in vegetation types with large differences in elevation distribution. This suggests that large changes in elevation increase macrofungal community turnover.

## 5. Conclusions

This study revealed the macrofungal diversity, community composition, and the distribution pattern (hump-shaped pattern) of the Shaluli Mountains, which has a high altitude and distinct vertical distribution. The elevation of vegetation types or tree species was the main factor influencing the distribution pattern. These findings provide a theoretical basis for the scientific conservation of macrofungal resources. Of course, our study had some limitations, and high-throughput soil sequencing combined with long-term collection of macrofungal specimens may provide a better explanation.

## Figures and Tables

**Table 1:** Composition of the macrofungal flora in different vegetation types.

Phylum	Class	Order	Family	Genus	Vegetation Type
Ascomycota	Geoglossomycetes	Geoglossales	Geoglossaceae	*Geoglossum*	Jun
				*Trichoglossum*	PQ
	Leotiomycetes	Helotiales	Chlorociboriaceae	*Chlorociboria*	PQ
			Helotiaceae	*Bisporella*	Pic
			Leotiaceae	*Leotia*	Pic, PQ, Que
	Pezizomycetes	Pezizales	Discinaceae	*Gyromitra*	Pin, Pop
			Helvellaceae	*Helvella*	Abi, PA, Pic, PQ, Que
			Otideaceae	*Otidea*	Abi, PA, Que
			Pyronemataceae	*Cheilymenia*	Pin
				*Humaria*	Pic, Pin, Que
				*Sowerbyella*	Que
			Sarcoscyphaceae	*Cookeina*	Pic
			Sarcosomataceae	*Plectania*	Que
			incertae sedis	*Tarzetta*	Que
		Rhytismatales	Cudoniaceae	*Cudonia*	PA, Pin
				*Spathularia*	Abi, Jun, PA, Pic, Pin
	Sordariomycetes	Hypocreales	Hypocreaceae	*Hypomyces*	Que
			Ophiocordycipitaceae	*Tolypocladium*	Pop
		Xylariales	Hypoxylaceae	*Daldinia*	Abi, Pic, Que
				*Hypoxylon*	Que
Basidiomycota	Agaricomycetes	Agaricales	Agaricaceae	*Agaricus*	AM, PA, Pic, Pin, PQ, Que
				*Holocotylon*	AM
				*Lepiota*	Abi, Pic, Que, SS
				*Leucoagaricus*	Abi
				*Leucocoprinus*	Abi
			Amanitaceae	*Amanita*	AM, PA, Pic, Pin, PQ, Que, SS
			Biannulariaceae	*Catathelasma*	PA, Pic
			Bolbitiaceae	*Pholiotina*	Pin
			Cortinariaceae	*Cortinarius*	Abi, AM, PA, Pic, Pin, Pop, PQ, Que, SS
			Crepidotaceae	*Crepidotus*	Pic
				*Pleuroflammula*	Pin
			Cyphellaceae	*Chondrostereum*	Pin
			Entolomataceae	*Clitopilus*	Abi, PA, Pop, Que
				*Entoloma*	Abi, AM, Jun, PA, Pic, PQ, Que, SS
			Hydnangiaceae	*Laccaria*	Abi, AM, PA, Pic, Pin, Pop, PQ, Que
			Hygrophoraceae	*Cuphophyllus*	Abi, Jun, Pop, Que, SS
				*Hygrocybe*	Abi, Jun, Pic, Que, SS, SS
				*Hygrophorus*	Abi, PA, Pic, Pin, PQ, Que
				*Lichenomphalia*	Abi, Pic, Pin
				*Spodocybe*	Pic
			Hymenogastraceae	*Galerina*	PA, Pin
				*Gymnopilus*	Abi, Pin
				*Hebeloma*	Abi, Jun, PA, Pic, Pin, Pop, Que, SS
				*incertae sedis*	Abi
			Inocybaceae	*Inocybe*	Abi, AM, Jun, PA, Pic, Pin, Pop, PQ, Que, SS
				*Inosperma*	Que
				*Mallocybe*	Abi, PA, Pop
				*Pseudosperma*	Que
			Lycoperdaceae	*Apioperdon*	Pic, Que
				*Bovista*	Abi, AM, Jun
				*Calvatia*	PA, Que
				*Lycoperdon*	Abi, AM, Jun, PA, Pic, Pin, Pop, Que, SS
			Lyophyllaceae	*Calocybe*	Pic
				*Hypsizygus*	Que
				*Lyophyllum*	Jun, PA, PQ, Que, SS
				*Tephrocybe*	PA
			Marasmiaceae	*Marasmius*	Abi, Jun, Pic
			Mycenaceae	*Hydropus*	Pic, Que
				*Mycena*	Abi, AM, PA, Pic, Pin, Pop, PQ, Que
				*Panellus*	Pic
				*Xeromphalina*	Pic, Pin
			Omphalotaceae	*Gymnopus*	Abi, AM, PA, Pic, Pin, PQ, Que
				*Lentinula*	Pin
				*Marasmiellus*	PQ, Que
				*Omphalotus*	PA
				*Rhodocollybia*	Abi
			Physalacriaceae	*Armillaria*	Abi, PA, Pic, Pin, Pop, Que
				*Hymenopellis*	Pin
				*Mucidula*	PQ
				*Oudemansiella*	PQ
				*Xerula*	Pic
			Pleurotaceae	*Hohenbuehelia*	Pic
				*Pleurotus*	Abi, PA, Pic
			Pluteaceae	*Pluteus*	Abi, PA, Pin, Pop, Que
				*Volvopluteus*	PA, PQ, Que
			Psathyrellaceae	*Coprinellus*	Que
				*Coprinopsis*	Que
				*Homophron*	Abi, PA
				*Psathyrella*	Pic, Que
			Pseudoclitocybaceae	*Pseudoclitocybe*	Que
			Strophariaceae	*Agrocybe*	PA
				*Deconica*	Pic
				*Hypholoma*	Pop
				*Pholiota*	Abi, Pic, Pin, Pop, Que
				*Protostropharia*	PA, PQ, Que
				*Stropharia*	AM, PA
			Tricholomataceae	*Leucopaxillus*	Pop
				*Pseudotricholoma*	Jun
				*Tricholoma*	Abi, Jun, PA, Pic, Pin, Que
			incertae sedis	*Aspropaxillus*	PA
				*Clitocybe*	Jun, PA, Pic, Que
				*Clitocybula*	Abi
				*Collybia*	PA, Pin, PQ, Que
				*Cyathus*	Pop
				*Cystoderma*	AM
				*Cystodermella*	Pic
				*Floccularia*	Abi, Jun, PA, Pin
				*Gerronema*	Pic
				*Infundibulicybe*	PA, Pic, Que
				*Lepista*	Abi, PA, Que
				*Melanoleuca*	Pic, Que
				*Mycenella*	Que
				*Nidula*	Pic
				*Panaeolus*	AM, Pic
				*Rhizocybe*	Pop, PQ
				*Tricholomopsis*	Pin, Pop
		Auriculariales	Auriculariaceae	*Auricularia*	Abi, Pic, Pop
			incertae sedis	*Guepinia*	PA, PQ
				*Ovipoculum*	Abi
		Boletales	Boletaceae	*Boletus*	Abi, PA, Que
				*Cyanoboletus*	Que
				*Harrya*	PQ
				*Hourangia*	Que
				*Leccinum*	Abi, AM, PA, Pin, Pop, Que, SS
				*Strobilomyces*	Que
				*Suillellus*	Que
				*Tylopilus*	Que
				*Xanthoconium*	Abi
				*Xerocomellus*	Abi, Que
				*Xerocomus*	PQ, Que
				*Zangia*	Que
			Gomphidiaceae	*Gomphidius*	Pic
			Paxillaceae	*Paxillus*	Que
			Rhizopogonaceae	*Rhizopogon*	Abi, Pin
			Sclerodermataceae	*Scleroderma*	PA, Que
			Suillaceae	*Suillus*	Abi, Pic, Pin
			Tapinellaceae	*Pseudomerulius*	Pic
				*Tapinella*	Pop
		Cantharellales	Hydnaceae	*Cantharellus*	Que
				*Clavulina*	Pic
				*Craterellus*	PQ
				*Hydnum*	Abi, Pic, Pin, PQ
		Geastrales	Geastraceae	*Geastrum*	PA
		Gomphales	Clavariadelphaceae	*Clavariadelphus*	Abi, PA, Pic, Pop, Que
			Gomphaceae	*Gomphus*	PA, Pic, Que, SS
				*Phaeoclavulina*	Jun, Pic, PQ, Que
				*Ramaria*	Abi, PA, Pic, Pin, PQ, Que
				*Turbinellus*	Pic, PQ
		Hymenochaetales	Hymenochaetaceae	*Coltricia*	Abi, PA
				*Inonotus*	Pin
				*Phellinus*	Que
			Rickenellaceae	*Cotylidia*	Pic
		Hysterangiales	Hysterangiaceae	*Hysterangium*	Pic
		Polyporales	Dacryobolaceae	*Amaropostia*	Pic
			Fomitopsidaceae	*Antrodia*	Pop
				*Fomitopsis*	Que
			Incrustoporiaceae	*Tyromyces*	Que
			Panaceae	*Panus*	PA
			Podoscyphaceae	*Abortiporus*	Que
			Polyporaceae	*Daedaleopsis*	Abi, PA, Pic, Pop
				*Ganoderma*	Que
				*Laccocephalum*	Abi
				*Lenzites*	Pic
				*Neofavolus*	Que
				*Polyporus*	PA, Pic, Pin, Pop, Que
				*Trametes*	Abi, Que
			Steccherinaceae	*Nigroporus*	Que
			incertae sedis	*Mycoleptodonoides*	Pic
		Russulales	Albatrellaceae	*Albatrellus*	PA, PQ
			Auriscalpiaceae	*Auriscalpium*	Abi
				*Lentinellus*	Pic
			Bondarzewiaceae	*Heterobasidion*	Pic, Pop
			Hericiaceae	*Hericium*	Que
			Russulaceae	*Lactarius*	Abi, AM, PA, Pic, Pin, Pop, PQ, Que, SS
				*Lactifluus*	Abi, Pop
				*Russula*	Abi, AM, Jun, PA, Pic, Pin, Pop, PQ, Que, SS
			Stereaceae	*Stereum*	PQ, Que
		Stereopsidales	Stereopsidaceae	*Stereopsis*	Abi, Pic, PQ
		Thelephorales	Bankeraceae	*Bankera*	Pic, Pin
				*Boletopsis*	PQ
				*Hydnellum*	Pic, Que
				*Sarcodon*	PA, Pin, Que
			Thelephoraceae	*Phellodon*	Pin
	Dacrymycetes	Dacrymycetales	Dacrymycetaceae	*Calocera*	Abi, PA, Pic, Pin
				*Dacrymyces*	Abi, Pic, Pop
				*Guepiniopsis*	Que
	Exobasidiomycetes	Exobasidiales	Exobasidiaceae	*Exobasidium*	Pic
	Tremellomycetes	Tremellales	Naemateliaceae	*Naematelia*	Que

**Table 2:** Dominant families (≥10 species) of macrofungi in Shaluli Mountains.

Family	Number of Species	Percentage (%)
Cortinariaceae	116	15.14%
Russulaceae	97	12.66%
Inocybaceae	56	7.31%
Hygrophoraceae	37	4.83%
Boletaceae	32	4.18%
Tricholomataceae	28	3.66%
Entolomataceae	25	3.26%
Mycenaceae	23	3.00%
Amanitaceae	22	2.87%
Hymenogastraceae	22	2.87%
Hydnangiaceae	20	2.61%
Lycoperdaceae	19	2.48%
Gomphaceae	16	2.09%
Omphalotaceae	15	1.96%
Strophariaceae	15	1.96%
Agaricaceae	14	1.83%
Polyporaceae	14	1.83%
Pluteaceae	10	1.31%
Lyophyllaceae	10	1.31%

**Table 3:** Dominant genera (≥10 species) of macrofungi in Shaluli Mountains.

Genra	Number of Species	Percentage (%)
*Cortinarius*	116	15.14%
*Russula*	59	7.70%
*Inocybe*	51	6.66%
*Lactarius*	37	4.83%
*Tricholoma*	26	3.39%
*Entoloma*	23	3.00%
*Amanita*	22	2.87%
*Laccaria*	20	2.61%
*Hygrophorus*	18	2.35%
*Mycena*	18	2.35%
*Lycoperdon*	14	1.83%
*Hebeloma*	13	1.70%
*Ramaria*	11	1.44%
*Hygrocybe*	10	1.31%
*Leccinum*	10	1.31%
*Gymnopus*	10	1.31%

## Data Availability

Not applicable.

## Supplementary Materials

The following supporting information can be downloaded at: https://www.mdpi.com/article/10.3390/jof9040491/s1, Table S1: List of macrofungi in the Shaluli Mountains.
